# 1-Isobutyl-*N*,*N*-dimethyl-1*H*-imidazo[4,5-*c*]quinolin-4-amine

**DOI:** 10.1107/S160053681100153X

**Published:** 2011-01-15

**Authors:** Wan-Sin Loh, Hoong-Kun Fun, Reshma Kayarmar, S. Viveka, G. K. Nagaraja

**Affiliations:** a X-ray Crystallography Unit, School of Physics, Universiti Sains Malaysia, 11800 USM, Penang, Malaysia; b Department of Chemistry, Mangalore University, Karnataka, India

## Abstract

In the title compound, C_16_H_20_N_4_, the 1*H*-imidazo[4,5-*c*]quinoline ring system is approximately planar, with a maximum deviation of 0.0719 (15) Å. An intra­molecular C—H⋯N hydrogen bond contributes to the stabilization of the mol­ecule, forming an *S*(6) ring motif. In the crystal, the mol­ecules are stacked along the *b* axis through weak aromatic π–π inter­actions between benzene and imidazole and benzene and pyridine rings [centroid–centroid distances = 3.6055 (10) and 3.5342 (10) Å, respectively].

## Related literature

For background to quinolines and their microbial activity, see: Jampilek *et al.* (2005 ▶); Gershon *et al.* (2004 ▶); Dardari *et al.* (2004 ▶). For the syntheses of 1*H*-imidazo[4,5-*c*]quinolin-4-amines, see: Gabriel (1918 ▶); Izumi *et al.* (2003 ▶). For bond-length data, see: Allen *et al.* (1987 ▶). For hydrogen-bond motifs, see: Bernstein *et al.* (1995 ▶).
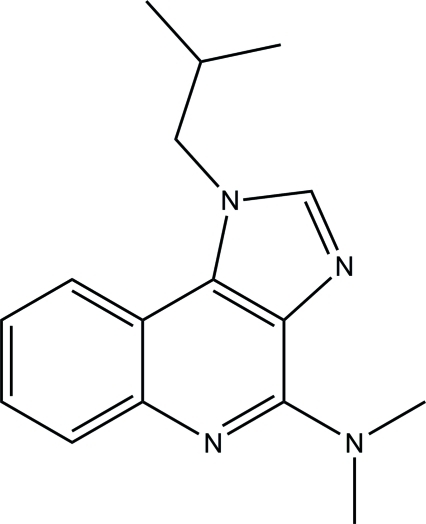


## Experimental

### 

#### Crystal data

C_16_H_20_N_4_
*M* *_r_* = 268.36
Monoclinic, 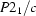

*a* = 9.2804 (2) Å
*b* = 18.5492 (6) Å
*c* = 8.5147 (2) Å
β = 101.051 (2)°
*V* = 1438.57 (7) Å^3^
*Z* = 4
Mo *K*α radiation
μ = 0.08 mm^−1^
*T* = 296 K
0.39 × 0.29 × 0.14 mm

#### Data collection

Bruker SMART APEXII CCD area-detector diffractometer
Absorption correction: multi-scan (*SADABS*; Bruker, 2009 ▶) *T*_min_ = 0.971, *T*_max_ = 0.989
13134 measured reflections
3456 independent reflections
2271 reflections with *I* > 2σ(*I*)
*R*_int_ = 0.037

#### Refinement

*R*[*F* ^2^ > 2σ(*F* ^2^)] = 0.055
*wR*(*F* ^2^) = 0.146
*S* = 1.02
3456 reflections
185 parameters
H-atom parameters constrained
Δρ_max_ = 0.26 e Å^−3^
Δρ_min_ = −0.18 e Å^−3^

### 

Data collection: *APEX2* (Bruker, 2009 ▶); cell refinement: *SAINT* (Bruker, 2009 ▶); data reduction: *SAINT*; program(s) used to solve structure: *SHELXTL* (Sheldrick, 2008 ▶); program(s) used to refine structure: *SHELXTL*; molecular graphics: *SHELXTL*; software used to prepare material for publication: *SHELXTL* and *PLATON* (Spek, 2009 ▶).

## Supplementary Material

Crystal structure: contains datablocks global, I. DOI: 10.1107/S160053681100153X/is2656sup1.cif

Structure factors: contains datablocks I. DOI: 10.1107/S160053681100153X/is2656Isup2.hkl

Additional supplementary materials:  crystallographic information; 3D view; checkCIF report

## Figures and Tables

**Table 1:** Hydrogen-bond geometry (Å, °)

*D*—H⋯*A*	*D*—H	H⋯*A*	*D*⋯*A*	*D*—H⋯*A*
C15—H15*A*⋯N3	0.96	2.16	2.918 (3)	135

**Fig. 1.**
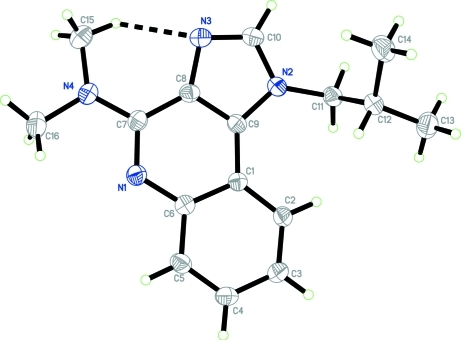
The molecular structure of the title compound, showing 30% probability displacement ellipsoids and the atom-numbering scheme. The dashed line indicates the intramolecular hydrogen bond.

**Fig. 2.**
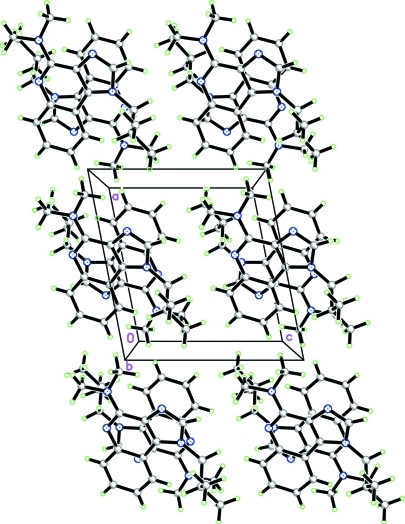
The crystal packing of the title compound, viewed along the b axis.

## supplementary crystallographic information

### Comment

Compounds bearing a quinoline moiety are well known due to their broad biological activity (Jampilek *et al.*, 2005). For example, hydroxyquinoline and its derivatives were introduced as antifungal agents in clinical practice and the novel compounds of this type are still being investigated (Gershon *et al.*, 2004; Dardari *et al.*, 2004). 1*H*-imidazo[4,5-*c*]quinolin-4-amines were synthesized by using two main synthetic routes. The first route started with 4-hydroxy-3-nitro-1*H*-quinolin-2-one, employing a modification of the method of Gabriel (Gabriel, 1918) to give 2,4-dichloro-3-nitroquinoline. Alternatively, the chlorination can be accomplished using phenylphosphonicdichloride (Izumi *et al.*, 2003). Reaction of the N-oxide with POCl_3_ in dichloromethane gave the 4-chloro-1*H*-imidazo[4,5-*c*]quinoline analogue, which was converted to 1-(2-methylpropyl)-1*H*-imidazo[4,5-*c*]quinolin-4-amine by treatment with NH_3_ in methanol at 150 °C. 1*H*-Imidazo-[4,5-*c*]quinolines are potential antiviral agents and also induce the production of cytokines, especially interferon (IFN). This promoted us to react 4-chloro-1-(2-methylpropyl)-1*H*-imidazo[4,5-*c*]quinolone with dimethylformide to give 1-isobutyl-*N,N*-dimethyl-1*H*-imidazo[4,5-*c*]quinolin-4-amine.

In the title compound (Fig. 1), the 1*H*-imidazo[4,5-*c*]quinoline ring system (C1–C6/N1/C7/C8/N3/C10/N2/C9) is approximately planar with a maximum deviation of 0.0719 (15) Å at atom N3. The torsion angle, C10—N2—C11—C12, formed between this ring system and the isobutyl unit is 100.8 (2)°. An intramolecular C15—H15A···N3 hydrogen bond (Table 1) contributes to the stabilization of the molecule, forming an *S*(6) ring motif (Bernstein *et al.*, 1995). Bond lengths (Allen *et al.*, 1987) and angles are within the normal ranges.

There is no significant intermolecular hydrogen bond observed in the crystal packing (Fig. 2). The molecules are stacked along the *b* axis by way of weak aromatic π–π interactions of the benzene C1–C6 ring (centroid *Cg*3) with the imidazole N2/C9/C8/N3/C10 (centroid *Cg*1) and pyridine N1/C6/C1/C9/C8/C7 (centroid *Cg*2) rings [*Cg*3···*Cg*1 separation = 3.6055 (10) Å; *Cg*3···*Cg*2 separation = 3.5342 (10) Å].

### Experimental

4-Chloro-1-(2-methylpropyl)-1*H*-imidazo[4,5-*c*]quinolone (2 g, 0.00771 mole), methanol (30 ml) and 3.3 ml of DMF were heated to reflux for 72 h. The solid formed was separated, filtered off and washed with methanol. Yield, 1.99 g (58.5%). Crystals suitable for *x*-ray analysis were obtained from ethanol by slow evaporation.

### Refinement

All H atoms were positioned geometrically (C—H = 0.93 to 0.98 Å) and refined using the riding model with *U*_iso_(H) = 1.2 or 1.5 *U*_eq_(C). A rotating group model was applied to the methyl groups.

### Crystal data

**Table:**

C_16_H_20_N_4_	*F*(000) = 576
*M**_r_* = 268.36	*D*_x_ = 1.239 Mg m^−^^3^
Monoclinic, *P*2_1_/*c*	Mo *K*α radiation, λ = 0.71073 Å
Hall symbol: -P 2ybc	Cell parameters from 3031 reflections
*a* = 9.2804 (2) Å	θ = 2.2–27.4°
*b* = 18.5492 (6) Å	µ = 0.08 mm^−^^1^
*c* = 8.5147 (2) Å	*T* = 296 K
β = 101.051 (2)°	Block, colourless
*V* = 1438.57 (7) Å^3^	0.39 × 0.29 × 0.14 mm
*Z* = 4	

### Data collection

**Table:**

Bruker SMART APEXII CCD area-detector diffractometer	3456 independent reflections
Radiation source: fine-focus sealed tube	2271 reflections with *I* > 2σ(*I*)
graphite	*R*_int_ = 0.037
φ and ω scans	θ_max_ = 28.0°, θ_min_ = 2.2°
Absorption correction: multi-scan (*SADABS*; Bruker, 2009)	*h* = −12→12
*T*_min_ = 0.971, *T*_max_ = 0.989	*k* = −19→24
13134 measured reflections	*l* = −10→11

### Refinement

**Table:**

Refinement on *F*^2^	Primary atom site location: structure-invariant direct methods
Least-squares matrix: full	Secondary atom site location: difference Fourier map
*R*[*F*^2^ > 2σ(*F*^2^)] = 0.055	Hydrogen site location: inferred from neighbouring sites
*wR*(*F*^2^) = 0.146	H-atom parameters constrained
*S* = 1.02	*w* = 1/[σ^2^(*F*_o_^2^) + (0.0604*P*)^2^ + 0.3608*P*] where *P* = (*F*_o_^2^ + 2*F*_c_^2^)/3
3456 reflections	(Δ/σ)_max_ = 0.001
185 parameters	Δρ_max_ = 0.26 e Å^−^^3^
0 restraints	Δρ_min_ = −0.18 e Å^−^^3^

### Special details

**Table:**

Geometry. All e.s.d.'s (except the e.s.d. in the dihedral angle between two l.s. planes) are estimated using the full covariance matrix. The cell e.s.d.'s are taken into account individually in the estimation of e.s.d.'s in distances, angles and torsion angles; correlations between e.s.d.'s in cell parameters are only used when they are defined by crystal symmetry. An approximate (isotropic) treatment of cell e.s.d.'s is used for estimating e.s.d.'s involving l.s. planes.
Refinement. Refinement of *F*^2^ against ALL reflections. The weighted *R*-factor *wR* and goodness of fit *S* are based on *F*^2^, conventional *R*-factors *R* are based on *F*, with *F* set to zero for negative *F*^2^. The threshold expression of *F*^2^ > σ(*F*^2^) is used only for calculating *R*-factors(gt) *etc*. and is not relevant to the choice of reflections for refinement. *R*-factors based on *F*^2^ are statistically about twice as large as those based on *F*, and *R*- factors based on ALL data will be even larger.

### Fractional atomic coordinates and isotropic or equivalent isotropic displacement parameters (Å^2^)

**Table:**

	*x*	*y*	*z*	*U*_iso_*/*U*_eq_	
N1	0.44302 (15)	0.08084 (8)	0.74563 (17)	0.0430 (4)	
N3	0.31258 (17)	0.18631 (8)	0.3740 (2)	0.0512 (4)	
N2	0.51431 (15)	0.14182 (8)	0.30069 (17)	0.0418 (4)	
N4	0.22238 (16)	0.14021 (9)	0.68830 (19)	0.0520 (4)	
C7	0.35046 (18)	0.12102 (9)	0.6440 (2)	0.0400 (4)	
C6	0.57198 (17)	0.05715 (9)	0.7062 (2)	0.0386 (4)	
C5	0.66567 (19)	0.01562 (10)	0.8208 (2)	0.0467 (5)	
H5A	0.6387	0.0059	0.9184	0.056*	
C4	0.7957 (2)	−0.01084 (11)	0.7918 (2)	0.0500 (5)	
H4A	0.8563	−0.0378	0.8698	0.060*	
C3	0.8376 (2)	0.00247 (10)	0.6460 (2)	0.0499 (5)	
H3A	0.9262	−0.0155	0.6269	0.060*	
C2	0.74870 (18)	0.04200 (9)	0.5307 (2)	0.0428 (4)	
H2A	0.7771	0.0500	0.4333	0.051*	
C1	0.61517 (17)	0.07070 (8)	0.55699 (19)	0.0358 (4)	
C9	0.51360 (17)	0.11474 (8)	0.45202 (19)	0.0363 (4)	
C8	0.38711 (18)	0.14192 (9)	0.4938 (2)	0.0395 (4)	
C10	0.3921 (2)	0.18403 (10)	0.2632 (2)	0.0510 (5)	
H10A	0.3677	0.2090	0.1670	0.061*	
C11	0.61699 (19)	0.12704 (10)	0.1942 (2)	0.0449 (4)	
H11A	0.5682	0.1362	0.0848	0.054*	
H11B	0.6434	0.0764	0.2024	0.054*	
C12	0.7563 (2)	0.17189 (10)	0.2307 (2)	0.0466 (4)	
H12A	0.7980	0.1669	0.3450	0.056*	
C13	0.8672 (2)	0.14245 (12)	0.1367 (3)	0.0684 (6)	
H13A	0.8840	0.0923	0.1609	0.103*	
H13B	0.9579	0.1684	0.1657	0.103*	
H13C	0.8298	0.1481	0.0242	0.103*	
C14	0.7246 (3)	0.25132 (11)	0.1958 (3)	0.0689 (6)	
H14A	0.8137	0.2785	0.2254	0.103*	
H14B	0.6539	0.2680	0.2564	0.103*	
H14C	0.6862	0.2576	0.0837	0.103*	
C15	0.1048 (2)	0.17851 (13)	0.5909 (3)	0.0690 (6)	
H15A	0.1212	0.1807	0.4830	0.103*	
H15B	0.1002	0.2265	0.6318	0.103*	
H15C	0.0139	0.1540	0.5923	0.103*	
C16	0.1954 (2)	0.11825 (15)	0.8435 (3)	0.0716 (7)	
H16A	0.2865	0.1175	0.9193	0.107*	
H16B	0.1527	0.0709	0.8355	0.107*	
H16C	0.1292	0.1517	0.8784	0.107*	

### Atomic displacement parameters (Å^2^)

**Table:**

	*U*^11^	*U*^22^	*U*^33^	*U*^12^	*U*^13^	*U*^23^
N1	0.0436 (8)	0.0471 (8)	0.0387 (8)	0.0035 (7)	0.0084 (6)	−0.0017 (7)
N3	0.0483 (8)	0.0502 (9)	0.0556 (10)	0.0094 (7)	0.0110 (7)	0.0128 (8)
N2	0.0434 (8)	0.0424 (8)	0.0401 (8)	−0.0001 (6)	0.0093 (6)	0.0054 (7)
N4	0.0468 (8)	0.0622 (10)	0.0497 (10)	0.0129 (8)	0.0161 (7)	0.0038 (8)
C7	0.0396 (9)	0.0384 (9)	0.0425 (10)	−0.0003 (7)	0.0089 (7)	−0.0055 (8)
C6	0.0388 (8)	0.0368 (9)	0.0395 (9)	−0.0017 (7)	0.0058 (7)	−0.0033 (7)
C5	0.0525 (11)	0.0512 (11)	0.0354 (10)	0.0042 (9)	0.0062 (8)	0.0028 (8)
C4	0.0479 (10)	0.0540 (11)	0.0456 (11)	0.0095 (9)	0.0023 (8)	0.0075 (9)
C3	0.0428 (10)	0.0512 (11)	0.0568 (12)	0.0086 (8)	0.0126 (8)	0.0068 (9)
C2	0.0446 (9)	0.0405 (9)	0.0453 (10)	0.0015 (8)	0.0137 (8)	0.0049 (8)
C1	0.0376 (8)	0.0309 (8)	0.0385 (9)	−0.0022 (7)	0.0064 (7)	−0.0010 (7)
C9	0.0410 (9)	0.0311 (8)	0.0367 (9)	−0.0036 (7)	0.0069 (7)	0.0000 (7)
C8	0.0400 (9)	0.0342 (8)	0.0432 (10)	0.0001 (7)	0.0052 (7)	0.0016 (8)
C10	0.0502 (10)	0.0514 (11)	0.0508 (11)	0.0071 (9)	0.0080 (9)	0.0145 (9)
C11	0.0500 (10)	0.0485 (10)	0.0368 (10)	0.0018 (8)	0.0100 (8)	0.0018 (8)
C12	0.0483 (10)	0.0483 (10)	0.0449 (10)	0.0000 (8)	0.0132 (8)	0.0083 (8)
C13	0.0610 (13)	0.0689 (14)	0.0824 (16)	0.0028 (11)	0.0312 (12)	0.0030 (13)
C14	0.0788 (15)	0.0514 (12)	0.0844 (17)	−0.0004 (11)	0.0352 (13)	0.0129 (12)
C15	0.0460 (11)	0.0844 (16)	0.0790 (16)	0.0166 (11)	0.0183 (10)	0.0156 (13)
C16	0.0606 (13)	0.1034 (19)	0.0566 (13)	0.0146 (12)	0.0256 (11)	0.0048 (13)

### Geometric parameters (Å, °)

**Table:**

N1—C7	1.325 (2)	C1—C9	1.425 (2)
N1—C6	1.376 (2)	C9—C8	1.385 (2)
N3—C10	1.305 (2)	C10—H10A	0.9300
N3—C8	1.388 (2)	C11—C12	1.519 (3)
N2—C10	1.365 (2)	C11—H11A	0.9700
N2—C9	1.384 (2)	C11—H11B	0.9700
N2—C11	1.461 (2)	C12—C14	1.521 (3)
N4—C7	1.361 (2)	C12—C13	1.521 (3)
N4—C15	1.427 (2)	C12—H12A	0.9800
N4—C16	1.450 (2)	C13—H13A	0.9600
C7—C8	1.439 (2)	C13—H13B	0.9600
C6—C5	1.406 (2)	C13—H13C	0.9600
C6—C1	1.426 (2)	C14—H14A	0.9600
C5—C4	1.368 (2)	C14—H14B	0.9600
C5—H5A	0.9300	C14—H14C	0.9600
C4—C3	1.393 (3)	C15—H15A	0.9600
C4—H4A	0.9300	C15—H15B	0.9600
C3—C2	1.368 (2)	C15—H15C	0.9600
C3—H3A	0.9300	C16—H16A	0.9600
C2—C1	1.405 (2)	C16—H16B	0.9600
C2—H2A	0.9300	C16—H16C	0.9600
			
C7—N1—C6	120.37 (15)	N2—C10—H10A	122.9
C10—N3—C8	103.93 (14)	N2—C11—C12	113.65 (15)
C10—N2—C9	105.91 (14)	N2—C11—H11A	108.8
C10—N2—C11	125.01 (15)	C12—C11—H11A	108.8
C9—N2—C11	129.00 (14)	N2—C11—H11B	108.8
C7—N4—C15	125.46 (16)	C12—C11—H11B	108.8
C7—N4—C16	119.42 (16)	H11A—C11—H11B	107.7
C15—N4—C16	115.06 (16)	C11—C12—C14	111.44 (16)
N1—C7—N4	117.22 (16)	C11—C12—C13	109.34 (16)
N1—C7—C8	119.85 (15)	C14—C12—C13	111.73 (17)
N4—C7—C8	122.93 (16)	C11—C12—H12A	108.1
N1—C6—C5	117.17 (15)	C14—C12—H12A	108.1
N1—C6—C1	124.58 (15)	C13—C12—H12A	108.1
C5—C6—C1	118.24 (15)	C12—C13—H13A	109.5
C4—C5—C6	121.37 (17)	C12—C13—H13B	109.5
C4—C5—H5A	119.3	H13A—C13—H13B	109.5
C6—C5—H5A	119.3	C12—C13—H13C	109.5
C5—C4—C3	120.26 (17)	H13A—C13—H13C	109.5
C5—C4—H4A	119.9	H13B—C13—H13C	109.5
C3—C4—H4A	119.9	C12—C14—H14A	109.5
C2—C3—C4	120.10 (17)	C12—C14—H14B	109.5
C2—C3—H3A	119.9	H14A—C14—H14B	109.5
C4—C3—H3A	119.9	C12—C14—H14C	109.5
C3—C2—C1	121.20 (16)	H14A—C14—H14C	109.5
C3—C2—H2A	119.4	H14B—C14—H14C	109.5
C1—C2—H2A	119.4	N4—C15—H15A	109.5
C2—C1—C9	127.93 (15)	N4—C15—H15B	109.5
C2—C1—C6	118.81 (15)	H15A—C15—H15B	109.5
C9—C1—C6	113.24 (14)	N4—C15—H15C	109.5
N2—C9—C8	105.21 (14)	H15A—C15—H15C	109.5
N2—C9—C1	132.15 (15)	H15B—C15—H15C	109.5
C8—C9—C1	122.60 (15)	N4—C16—H16A	109.5
C9—C8—N3	110.77 (15)	N4—C16—H16B	109.5
C9—C8—C7	119.13 (15)	H16A—C16—H16B	109.5
N3—C8—C7	130.09 (15)	N4—C16—H16C	109.5
N3—C10—N2	114.14 (16)	H16A—C16—H16C	109.5
N3—C10—H10A	122.9	H16B—C16—H16C	109.5
			
C6—N1—C7—N4	177.51 (15)	C11—N2—C9—C1	6.5 (3)
C6—N1—C7—C8	−2.0 (2)	C2—C1—C9—N2	−0.3 (3)
C15—N4—C7—N1	−175.07 (19)	C6—C1—C9—N2	178.26 (16)
C16—N4—C7—N1	1.9 (3)	C2—C1—C9—C8	−177.77 (16)
C15—N4—C7—C8	4.4 (3)	C6—C1—C9—C8	0.8 (2)
C16—N4—C7—C8	−178.62 (18)	N2—C9—C8—N3	−1.55 (19)
C7—N1—C6—C5	179.12 (15)	C1—C9—C8—N3	176.52 (15)
C7—N1—C6—C1	−2.1 (3)	N2—C9—C8—C7	177.37 (14)
N1—C6—C5—C4	179.43 (17)	C1—C9—C8—C7	−4.6 (2)
C1—C6—C5—C4	0.5 (3)	C10—N3—C8—C9	1.2 (2)
C6—C5—C4—C3	−0.6 (3)	C10—N3—C8—C7	−177.58 (18)
C5—C4—C3—C2	−0.1 (3)	N1—C7—C8—C9	5.2 (2)
C4—C3—C2—C1	0.8 (3)	N4—C7—C8—C9	−174.26 (16)
C3—C2—C1—C9	177.63 (17)	N1—C7—C8—N3	−176.11 (17)
C3—C2—C1—C6	−0.8 (3)	N4—C7—C8—N3	4.4 (3)
N1—C6—C1—C2	−178.65 (15)	C8—N3—C10—N2	−0.4 (2)
C5—C6—C1—C2	0.1 (2)	C9—N2—C10—N3	−0.6 (2)
N1—C6—C1—C9	2.7 (2)	C11—N2—C10—N3	176.48 (16)
C5—C6—C1—C9	−178.54 (15)	C10—N2—C11—C12	100.8 (2)
C10—N2—C9—C8	1.26 (18)	C9—N2—C11—C12	−82.9 (2)
C11—N2—C9—C8	−175.65 (16)	N2—C11—C12—C14	−67.1 (2)
C10—N2—C9—C1	−176.54 (18)	N2—C11—C12—C13	168.91 (16)

### Hydrogen-bond geometry (Å, °)

**Table:**

*D*—H···*A*	*D*—H	H···*A*	*D*···*A*	*D*—H···*A*
C15—H15A···N3	0.96	2.16	2.918 (3)	135
